# Ursodeoxycholic Acid Regulates Hepatic Energy Homeostasis and White Adipose Tissue Macrophages Polarization in Leptin-Deficiency Obese Mice

**DOI:** 10.3390/cells8030253

**Published:** 2019-03-16

**Authors:** Yu-Sheng Chen, Hsuan-Miao Liu, Tzung-Yan Lee

**Affiliations:** 1 Graduate Institute of Clinical Medical Science, College of Medicine, Chang Gung University, No. 259, Wen-Hwa 1st Road, Kwei-Shan, Taoyuan 333, Taiwan; whaich@msn.com; 2 Division of Chinese Acupuncture, Center for Traditional Chinese Medicine, Chang Gung Memorial Hospital, No. 123, Dinghu Road, Guishan District, Taoyuan 333, Taiwan; 3 Graduate Institute of Traditional Chinese Medicine, School of Chinese Medicine, College of Medicine, Chang Gung University, Taoyuan 33302, Taiwan; miaowhale@gmail.com; 4 Department of Traditional Chinese Medicine, Chang Gung Memorial Hospital, Keelung 204, Taiwan

**Keywords:** ursodeoxycholic acid, mitochondria, macrophage browning, obesity

## Abstract

Obesity has been shown to play a role in the pathogenesis of several forms of metabolic syndrome, including non-alcoholic fatty liver disease (NAFLD) and type 2 diabetes. Ursodeoxycholic acid (UDCA) has been shown to possess antioxidant and anti-inflammatory properties and prevents mitochondrial dysfunction in the progression of obesity-associated diseases. The aim of the study was to evaluate the mechanisms of UDCA during obesity-linked hepatic mitochondrial dysfunction and obesity-associated adipose tissue macrophage-induced inflammation in obese mice. UDCA significantly decreased lipid droplets, reduced free fatty acids (FFA) and triglycerides (TG), improved mitochondrial function, and enhanced white adipose tissue browning in *ob/ob* mice. This is associated with increased hepatic energy expenditure, mitochondria biogenesis, and incorporation of bile acid metabolism (Abca1, Abcg1 mRNA and BSEP, FGFR4, and TGR5 protein). In addition, UDCA downregulated NF-κB and STAT3 phosphorylation by negative regulation of the expression of SOCS1 and SOCS3 signaling. These changes were accompanied by decreased angiogenesis, as shown by the downregulation of VEGF, VCAM, and TGF-βRII expression. Importantly, UDCA is equally effective in reducing whole body adiposity. This is associated with decreased adipose tissue expression of macrophage infiltration (CD11b, CD163, and CD206) and lipogenic capacity markers (lipofuscin, SREBP-1, and CD36). Furthermore, UDCA significantly upregulated adipose browning in association with upregulation of SIRT-1-PGC1-α signaling in epididymis adipose tissue (EWAT). These results suggest that multi-targeted therapies modulate glucose and lipid biosynthesis fluxes, inflammatory response, angiogenesis, and macrophage differentiation. Therefore, it may be suggested that UDCA treatment may be a novel therapeutic agent for obesity.

## 1. Introduction

Obesity-induced non-alcoholic fatty liver disease (NAFLD) is a major risk factor for type 2 diabetes, hyperlipidemia, and some types of cancer [1,2]. Although the mechanism by which obesity causes NAFLD is unclear, inflammation has been linked to the development of local and systemic metabolic syndrome, especially, the inflammatory signaling occurs in liver and white adipose tissue (WAT) [3,4]. The mitochondria within the liver and WAT is the energy metabolism center and is maintained through a combination of fatty acid beta-oxidation and mitochondrial respiratory chain (MRC) activity that is regulated partly by adenosine triphosphate (ATP) needed for lipogenesis and gluconeogenesis [1,2,3]. Mitochondrial dysfunction, oxidation stress, high free fatty acids (HFFA), inflammatory response, and M1 macrophage polarization are observed in obesity-related diseases, including NAFLD, metabolic syndrome, and type 2 diabetes [4]. Obesity is characterized as a chronic state of low-grade inflammation with progressive immune cell infiltration into adipose tissues. Adipose tissue macrophages play critical roles in the establishment of the chronic inflammatory state and metabolic dysfunctions. Macrophages are classified in to two types: proinflammatory M1 and anti-inflammatory M2 Macrophages. M1 macrophages expresses on the surface markers cluster of differentiation (CD) 11c, C-C chemokine receptor type 7 (CCR7), and displays enhanced glycolytic metabolism and reduced mitochondrial activity [5]. M2 macrophages express on the surface markers CD206 and CD163 and show high mitochondrial oxidative phosphorylation (OXPHOS) and are characterized by an enhanced spare respiratory capacity (SRC) [5]. New insights reveal that a cell subpopulation of WAT, called beige or brite adipocytes, under alternative activation of macrophages (i.e., M2 polarization) contributes to express uncoupling protein 1 (UCP1) and are engaged in thermogenesis and browning of WAT (as called beige adipocytes), that provides a defense against cold and obesity [6,7,8,9]. Macrophages are central to immunometabolism, obesity-associated tissue remodeling, and the development of adiposity-based chronic systemic inflammation, metabolic syndrome, non-alcoholic fatty liver disease, and type 2 diabetes (T2D). Thus, understanding the mechanisms involved in the macrophage polarization is a key element in finding a novel therapeutic target for tracking obesity.

Ursodeoxycholic acid (UDCA) is a hydrophilic bile acid (BA) and has been administered occasionally as a hepatoprotective drug for cholestasis and chronic hepatitis; it is also the drug approved by the United States Food and Drug Administration for the treatment of primary biliary cholangitis (PBC) [10,11]. Early reports indicate that UDCA improves glucose metabolism; that is, administration of high-dose UDCA improves glycemic parameters, insulin sensitivity, and insulin resistance surrogate markers in patients with NASH [12,13]. Moreover, UDCA exerts choleretic [14], anti-apoptotic [15], and immunomodulation effects [16]; alternation of the bile acid composition [17] is also capable of enhancing the defenses against oxidative stress; and inhibition of apoptosis is induced by several agents [18]. Recent reports indicate that UDCA modulates multiple molecular targets and has potent anti-inflammatory activities and improves glucose and lipid metabolism [19], which may contribute to its therapeutic role in obesity and obesity-related metabolic diseases [18]. UDCA has shown to protect against alcohol-induced hepatotoxicity by improving mitochondrial function and attenuating oxidative stress in vivo [20]. However, the therapeutic use of UDCA is no significant reduced CD11b and F4/80 in sclerosing cholangitis in Mdr2 (Abcb4) knockout mice [21]. Although the incidence of obesity and related metabolic disorders continues to increase in modern society, there are no pharmacological therapies for its treatment. Thus, development of UDCA as a safe and effective therapy remains a focus attention to alleviate obesity. Therefore, this study was to determine if UDCA causes resistance to mitochondria dysfunction and macrophage polarization following the obese process. Three questions are addressed: (i) are overall obese mice body weight, total TG, and FFA levels ameliorated by the administration of UDCA (ii) is hepatic mitochondria dysfunction, inflammation, and angiogenesis attenuated by UDCA treatment; and (iii) a comparison of epididymis adipose tissue macrophage differentiation response is regulated by UDCA. These data demonstrate that UDCA plays a critical role in controlling liver and EWAT pathophysiology and energy homeostasis, and obesity leads to dysfunction causes by excessive fat accumulation. 

## 2. Materials and Methods

### 2.1. Cell Culture and Treatment

Alpha mouse liver 12 (AML12) cell line (passages 7–10, obtained from Bioresource Collection and Research Centre, Taiwan) was grown at 37 °C in Dulbecco’s modified Eagle’s medium/Ham’s nutrient mixture F-12 (DMEM/F12), 10% fetal bovine serum (FBS), 2 mmol/L l-Glutamine and 100 μg/mL penicillin and streptomycin (both from Gibco, AntiSel, Greece) at 5% CO_2_. Cells were starved in DMEM/F12 with 0.5% FBS for 12 h, followed by treatment with control medium 0.16 mmol/liter bovine serum albumin (BSA) and 1 mM HFFA medium (addition of 1.0 mmol/liter fatty acid mixture of 2:1 palmitate/oleate) with 0.16 mmol/liter BSA supplemented. This 1 mM HFFA construct was used in vitro to produce steatosis [22]. In vitro experiments were carried out in AML12 cells treated with 1 mM HFFA with 10, 30, 100 μM UDCA (100 mM stock solution in dimethyl sulfoxide, finally diluted 1:1000 in DMEM/F12; UDCA from Sigma-Aldrich, U5127).

### 2.2. ROS Activity Measurement

Cells were incubated in 6-well plates (2 × 10^5^ cells/well) with UDCA or DMSO control in 1 mM HFFA DMEM/F12 medium for 12 h [22]. Cultured AML12 cells were fixed in buffered formalin. For the quantitative analysis of ROS production, cells were incubated with 2′,7′-Dichlorofluorescin diacetate (DCFH-DA, Sigma-Aldrich; Merck KGaA, Darmstadt, Germany) for 30 min according to the manufacturer’s manuals [23], followed by treatment with DAPI (Thermo Fisher Scientific, Waltham, MA, USA), which was used for nuclear staining.

### 2.3. Mitochondria Mass Measurement

Cells were incubated in 6-well plates (2 × 10^5^ cells/ well) with UDCA or DMSO control in 1 mM HFFA DMEM/F12 medium for 12 h. Cultured AML12 cells were fixed in buffered formalin. For the quantitative analysis of mitochondria mass, cells were incubated with MitoTracker^®^ (Thermo Fisher Scientific). To label mitochondria, cells are simply incubated for 30 min at 37 °C with MitoTracker^®^ probes, which passively diffuse across the plasma membrane and accumulate in active mitochondria [24]. DAPI was used for nuclear staining. 

### 2.4. Animals 

Male C57BL/6J (normal) mice and leptin-deficient C57BL/6J (also named *ob/ob*) mice were obtained from the National Laboratory Animal Center (Taiwan). All animals were fed standard chow and water ad libitum and housed under conditions of control temperature (26 °C) and illumination (12-h light/dark cycle, light off at 7 pm). Twenty-week-old mice were randomized into three groups (n = 5 per group): (i) normal mice, (ii) *ob/ob* mice, and (iii) *ob/ob* mice were fed with UDCA (50 mg/kg/mouse, stock solution in 33% ethanol, 33% dimethyl sulfoxide, 33% Tween-80, finally diluted 1:50 in 0.5 M NaCl; UDCA from Sigma-Aldrich, U5127) once a day orally for 14 consecutive days. Each experimental group consisted of five animals and all mice remained on their assigned diet until they were sacrificed by CO_2_. All protocols were conducted in accordance with the Guide for the Care and Use of Laboratory Animals and were approved by the Chang Gung University Animal Care and Use Committee.

### 2.5. Histology, Immunohistochemistry and Immunofluorescence

Cultured AML12 cells were fixed in buffered formalin. Paraffin-embedded tissue was sectioned on a microtome (5 μm) and mounted on a microscope glass. Sections were deparaffinized. For histology, sections were attained with hematoxylin (2 g/L) for 15 min and with eosin (0.1%; in 0.0003% acetic acid) for 10 min. For immunofluorescence, epitopes were unmasked by the use of heat treatment in sodium citrate buffer (10 mM) at 95 °C for 15 min. Sections were preincubated with 5% non-immune goat serum and incubated with anti-SREBP-1c (Santz cruz Biotechnology, Inc., Santa Cruz, CA, USA), CD36 (Santz cruz), SIRT1 (Abcam, Cambridge, UK), PGC-1α (Abcam, UK), FXR (Santz cruz,), TLR4 (Abcam, UK), NF-κB (Abcam, UK), HIF-1α (Abcam, UK), NTCP (Abcam, UK), OATP1 (Abcam, USA), OSTβ (Biorbyt, San Francisco, CA, USA), BSEP (Abcam, UK), MRP3 (Abcam, UK), FGFR4 (Abcam, UK), TGR5 (Abcam, UK), neutrophil (Abcam, UK), F4/80 (BioLegend, San Diego, CA, USA), CD11b (Abcam, UK), CD11c (Abcam, UK), CCR7 (Abcam, UK), CD163 (Abcam, UK), CD206 (Abcam, UK), CD34 (Abcam, UK), CD90 (Abcam, UK), Tmem26 (Abcam, UK), UCP1 (Abcam, UK), α-SMA (Sigma, USA), TGFβ RII (Santz cruz), VEGF (Santz cruz), VEGFRI (Epitomics, Burlingame, CA, USA) and VCAM (Santz cruz) antibody at room temperature for 2 h. Sections were incubated with the secondary antibody (Alexa Fluor 488, Alexa Fluor 633, anti-mouse or anti-rabbit, Thermo Fisher Scientific) for 60 min at room temperature. All antibodies were diluted in 2% non-immune goat serum in PBST buffer. Sections were incubated with 3,3′-diaminobenzidine (DAB; Thermo Fisher Scientific) for 5 min, and DAPI (Thermo Fisher Scientific) or hematoxylin (Sigma, USA) was used for nuclear staining. Positive staining for CD11c, CCR7, CD163, and CD206 were quantified using ImageJ software (1.45, NIH). The antibodies used for immunohistochemistry and immunofluorescence are listed in Table 1.

### 2.6. Oil Red O Stain

Cells were incubated in 6-well plates with UDCA or DMSO control in 1 mM HFFA DMEM/F12 medium for 24 h. Cultured AML12 cells were fixed in buffered formalin. For the quantitative analysis of lipids, cells were incubated with Oil Red O (Sigma-Aldrich; Merck KGaA, Darmstadt, Germany). Fresh liver tissues were embedded carefully in an optimal cutting temperature compound (OCT) in a plastic mold, followed by freezing at −80 °C. Cells and liver tissue sections (10 μm thick) were stained with Oil Red O working solution (*w*/*v*, 60% isopropyl alcohol and 40% water) for 15 min. Cells and liver tissue sections were rinsed with 50% isopropanol and counterstained with hematoxylin for the nucleus. Fat droplets in the liver were stained red.

### 2.7. Auto-Fluorescence Detection of Lipofuscin

The liver and EWAT sections were deparaffinized, hydrated, and mounted into 40% glycerol/TBS mounting medium. Lipofuscin auto-fluorescence was then evidenced by excitation at 450–490 nm, using a dichromatic mirror at 510 nm and a long-pass filter at 515 nm [25]. In addition, DAPI was used for nuclear staining (Thermo Fisher Scientific).

### 2.8. Plasma Aminotransferase (ALT) Analysis

ALT measurements were used in the diagnosis and treatment of certain liver diseases. Plasma ALT was determined with the ALT assay kit (RANDOX, Antrim, United Kingdom).

### 2.9. Plasma and Hepatic Triglyceride (TG) Measurement

The plasma sample was used directly. We extracted the lipids from liver samples using chloroform/methanol (2:1) for 1 h at 25 °C, as described previously [26], and then analyzed hepatic TG with TG kit (RANDOX).

### 2.10. Western Blot Analysis

Liver and EWAT were harvested immediately, flash frozen in liquid nitrogen, and stored at −80 °C. Liver and EWAT were laced with distilled water containing protease inhibitors and a Bio-Rad Rapid Coomassie kit (Bio-Rad, Hercules, CA, USA) was used to determine the protein concentration. Protein (50 μg) was run on a 10% SDS-polyacrylamide gel and transferred to a polyvinylidene difluoride membrane. Immunoblotting was performed with various mouse/rabbit monoclonal or polyclonal antibodies, followed by the incubation of the appropriate secondary antibody coupled with horseradish peroxidase. The blot was developed with a chemiluminescence system (ECL; Amersham, Piscataway, NJ, USA) according to the manufacturer’s instructions. The optical densities of the bands were quantified using the GS-700 Imaging Densitometer (Bio-Rad). Antibodies: SREBP-1c (Santz cruz), CD36 (Santz cruz), Notch1(Abcam, UK), NICD (Abcam, UK), PEPCK (Santz cruz), SIRT1 (Abcam, UK), PGC-1α (Abcam, UK), G6Pase (Santz cruz), NF-κB (Abcam, UK), p-STAT3 (Millipore, Germany), STAT3 (Millipore, Germany), FXR (Santz cruz), CYP7A1 (Santz cruz), CD11c (Abcam, UK), CCR7 (Abcam, UK), CD163 (Abcam, UK), CD206 (Abcam, UK), Histone (Santz cruz), CD34 (Abcam, UK), CD90 (Abcam, UK), Tmem26 (Abcam, UK), UCP1 (Abcam, UK), c-Myc (Abcam, UK), α-SMA (Sigma, USA), β-catenin (Abcam, UK), TGFβ RII (Santz cruz), VEGF (Abcam, UK), VEGFRI (Epitomics), VCAM (Santz cruz), and β-actin (Millipore, Germany). All antibodies diluted in 1% skimmed milk in TTBS buffer. The protein expression was detected using an enhanced chemiluminescence kit (Millipore, USA-Bedford, MD, USA) and quantified using ImageQuant 5.2 software. The antibodies used for Western blot are listed in Table 1.

### 2.11. RNA Isolation and Real-Time PCR Analysis

Total RNA was isolated from tissues using TRzol and RNeasy kit (Thermo Fisher Scientific). Equal amounts of RNA were retrotranscribed to cDNA using High-Capacity cDNA Reverse transcription kit (Thermo Fisher Scientific). qRT-PCR (real-time reverse transcription-PCR) was run in 10 μL reactions in a Roche real-time PCR system machine from Applied Biosystems using SYBR Green PCR master mix (Roche, Schweizerische) according to the manufacturer’s instructions. Standard and melting curves were run in every plate for every gene to ensure efficiency and specificity of the reaction. The sequences of primers used for RT-qPCR are listed in Table 2.

### 2.12. Statistical Analysis

Data were presented as the means ± standard error of the mean (SEM). The statistical analyses were performed using Student’s *t*-test and one-way analysis of variance followed by the Student Newman-Keuls multiple-range test. A value of *P* < 0.05 was considered to indicate a statistically significant difference.

## 3. Results

### 3.1. UDCA Attenuates High Free Fatty Acid (HFFA) Induced-Lipid Accumulation and Mitochondrial Dysfunction of in AML12 Cells

**Figure 1:**
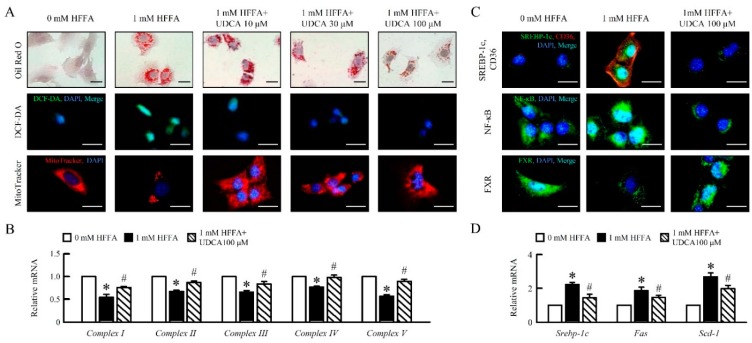
Ursodeoxycholic acid (UDCA) alleviates high free fatty acid (HFFA)-induced hepatocyte lipogenesis, reactive oxygen species (ROS) production, and mitochondrial dysfunction in AML12 cells. AML12 cells were treated with 1 mM HFFA with 10, 30, 100 μM UDCA. (**A**) Lipid accumulation display using Oil Red O stain (red). ROS levels were measured using DCFH-DA (green) stain. Images of AML12 cells stained with Mito Tracker for mitochondria (red). qRT-PCR analysis of (**B**) *Complex I, II, III, IV,* and *V* mRNA expression in AML12 cells. Relative mRNA expression was normalized to *Gapdh* and then normalized to the controls. (**C**) Immunofluorescence analysis of SREBP1c (green), CD36 (red), NF-κB (green), and FXR (green) expression, and DAPI (blue) for nuclear. Scale bar, 25 μm. qRT-PCR analysis of (**D**) *Srebp-1c, Fas*, and *Scd-1* mRNA expression in AML12 cells. In all panels, results are expressed as the mean ± S.E.M. of five independent experiments, and statistical significance of differences between means was assessed using an unpaired Student’s *t*-test (* *p* ≤ 0.05; 0 mM HFFA vs. 1 mM HFFA. ^#^ *p* ≤ 0.05; 1 mM HFFA vs. 1 mM HFFA+ 100 μM UDCA). UDCA, ursodeoxycholic acid; HFFA, high free fatty acid; ROS, reactive oxygen species; SREBP-1c, sterol regulatory element-binding protein-1c; CD36, cluster of differentiation 36; NF-κB, nuclear factor kappa-light-chain-enhancer of activated B cells; FXR, farnesoid X receptor; *Fas*, fatty acid synthase; *Scd-1,* stearoyl-CoA desaturase-1; qRT-PCR, quantitative real-time polymerase chain reaction; *Gapdh*, glyceraldehyde-3-phosphate dehydrogenase.

To determine the role of UDCA during HFFA treatment in AML12 cells, we analyzed the lipid accumulation, reactive oxygen species (ROS), and mitochondrial respiratory chain (MRC) mRNA expression in AML12 cells. UDCA attenuated HFFA-induced lipid accumulation, ROS generation, and improved mitochondrial swelling in AML12 (Figure 1A). UDCA significantly enhanced mitochondria *Complex I, II, III, IV* and *V* mRNA expression in AML12 cells treatment of HFFA (Figure 1B). Interestingly, fat depot-specific changes followed UDCA treatment. Accordingly, we observed that the administration of UDCA decreased sterol regulatory element-binding protein 1c (SREBP-1c), cluster of differentiation 36 (CD36), and nuclear factor kappa-light-chain-enhancer of activated B cells (NF-κB); improved farnesoid X receptor (FXR) expression (Figure 1C); and reduced *Srebp-1c*, fatty acid synthase (*Fas*), and stearoyl-CoA desaturase (*Scd*)-1 mRNA expression in AML12 cells treated HFFA (Figure 1D). Taken together, these results suggested that UDCA attenuated HFFA-induced lipid accumulation, ROS production, mitochondrial dysfunction, and inflammation in AML12 cells.

### 3.2. UDCA Attenuates Hepatosteatosis by Reducing Lipogenesis and β-Oxidation in ob/ob Mice

**Figure 2:**
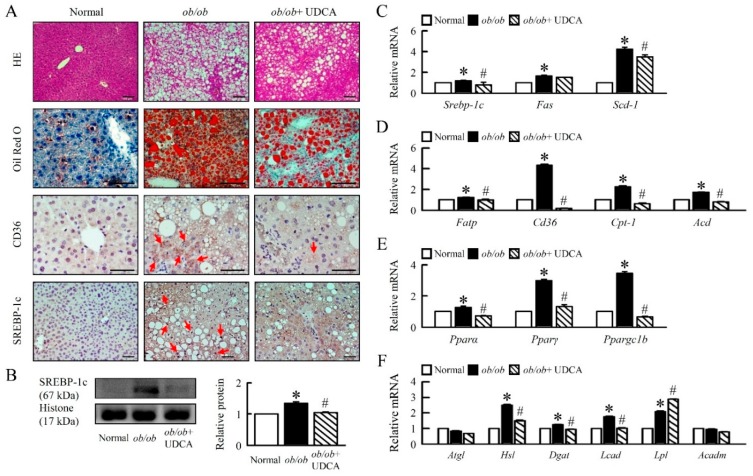
UDCA improves hepatic lipid metabolism disorders in *ob/ob* mice. Normal and *ob/ob* mice were treated with or without 50 mg/kg UDCA for 14 days. (**A**) Representative pictures of HE and Oil Red O staining of liver section. Immunohistochemical staining of CD36 and SREBP-1c in the liver. Red arrow highlights the positive staining. Scale bars, 100 µm. (**B**) Western blot analysis of SREBP-1c protein level in liver. qRT-PCR analysis of (**C**) *Srebp-1c, Fas, Scd-1,* (**D**) *Fatp, Cd36, Cpt-1, Aco,* (**E**) *Pparα, Pparγ, Ppargc1b*, (**F**) *Atgl, Hsl, Dgat, Lcad, Lpl*, and *Acadm* mRNA expression in liver. Relative mRNA expression was normalized to *Gapdh* and then normalized to the controls. In all panels, results are expressed as the mean ± S.E.M. of five independent experiments, and statistical significance of differences between means was assessed using an unpaired Student’s *t*-test (* *p* ≤ 0.05; normal vs. *ob/ob*. ^#^ *p* ≤ 0.05; *ob/ob* vs. *ob/ob* + UDCA). HE: hematoxylin and eosin; SREBP-1c, sterol regulatory element-binding proteins; *Fatp*, fatty acid transporter protein; *Cpt-1*, carnitine palmitoyltransferase 1; *Aco*, Acyl-CoA oxidase; *Ppar*, peroxisome proliferator-activated receptor; *Atg*, adipose triglyceride lipase; *Hsl*, hormone-sensitive lipase; *Dgat*, diacylglycerol acyltransferase; *Lcad*, long-chain Acyl-CoA dehydrogenase; *Lpl*, lipoprotein lipase; *Acadm*, medium-chain acyl-CoA dehydrogenase (MCAD).

*ob/ob* mice showed a high body weight compared with normal mice; we determined that UDCA significantly reduced body weight and improved the ratio of liver weight to body weight (Table 3). Plasma ALT, cholesterol, plasma and hepatic TG, and FFA levels were significantly decreased in *ob/ob* mice treated with UDCA (Table 3), suggesting UDCA ameliorated obesity and altered lipid metabolism. To further investigate the effects of UDCA on hepatosteatosis in *ob/ob* mice, we measured lipid deposits and the pathway of lipid metabolism in *ob/ob* mice. Histological sections of *ob/ob* liver showed large lipid-filled vacuoles, whereas *ob/ob* mice treatment UDCA showed a significantly smaller lipid-filled vacuole (Figure 2A). Immunohistochemical analysis showed the high CD36 and SREBP-1c protein levels in *ob/ob* mice, and high *Srebp-1c, Fas,* and *Scd-1* mRNA expression were found in liver tissue. We showed that UDCA significantly reduced the lipogenesis pathway in the liver (Figure 2A–C). UDCA attenuated fatty acid transporter protein (*Fatp*), *Cd36*, carnitine palmitoyltransferase 1 (*Cpt-1*), acyl-CoA oxidase (*Aco*), and decreased peroxisome proliferator-activated receptor (*Ppar*) α, *Pparγ*, PPARγ coactivator 1-beta (*Ppargc1b*), hormone-sensitive lipase (*Hsl*), diacylglycerol acyltransferase (*Dgat*), and long-chain Acyl-CoA dehydrogenase (*Lcad*) mRNA expression in *ob/ob* mice (Figure 2D–F). No changes were observed in the mRNA expression of adipose triglyceride lipase (*Atgl*) and acyl-coa dehydrogenase (*Acadm*) (Figure 2F). Obesity-induced lipoprotein lipase (*Lpl*) mRNA expression, and UDCA enhanced LPL expression in *ob/ob* mice (Figure 2F). Thus, UDCA is an important regulator of lipid metabolism. These features were paralleled by histological modifications.

### 3.3. UDCA Ameliorates Hepatic Glucose Metabolism Disorder and Notch1 Signaling in ob/ob Mice

**Figure 3:**
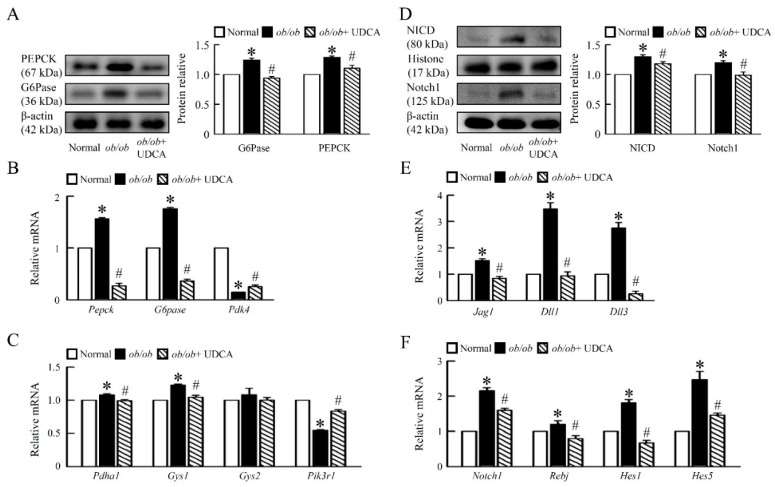
UDCA reduces hepatic Notch1 signaling and glucose metabolism disorders in *ob/ob* mice. Normal and *ob/ob* mice were treated with or without 50 mg/kg UDCA for 14 days. (**A**) Western blot analysis of PEPCK and G6Pase protein levels in liver. qRT-PCR analysis of (**B**) *Pepck, G6pase, Pdk4,* (**C**) *Pdha1, Gys1, Gys2*, and *Pik3r1* mRNA expression in liver. (**D**) Western blot analysis of NICD and Notch1 protein levels in liver. qRT-PCR analysis of (**E**) *Jag1, Dll1, Dll3*, (**F**) *Notch1, Rbpj, Hes1,* and *Hes5* mRNA expression in liver. Relative mRNA expression was normalized to *Gapdh* and then normalized to the controls. In all panels, results are expressed as the mean ± S.E.M. of five independent experiments, and statistical significance of differences between means was assessed using an unpaired Student’s *t*-test (* *p* ≤ 0.05; normal vs. *ob/ob*. ^#^ *p* ≤ 0.05; *ob/ob* vs. *ob/ob* + UDCA). PEPCK, phosphoenolpyruvate carboxykinase; G6Pase, glucose 6-phosphatase; *Pdk4*, pyruvate dehydrogenase kinase 4; *Pdha1*, pyruvate dehydrogenase E1 alpha 1; *Gys*, glycogen synthase; *Pik3r1*, phosphatidylinositol 3-kinase regulatory subunit 1; Notch1, neurogenic locus notch homolog protein 1; NICD, Notch intracellular domain; *Jag1*, Jagged1; *Dll*, delta like canonical Notch ligand; *Rbpj*, recombining binding protein suppressor of hairless; *Hes*, hairy and enhancer of split.

To determine the dynamics of recovery from obesity-associated glucose dysmetabolism, we identified the mechanisms of hepatic glucose metabolism in mice. Protein levels of phosphoenolpyruvate carboxykinase (PEPCK) and glucose-6-phosphatase (G6Pase) were increased in *ob/ob* mice, and the mRNA expression of pyruvate dehydrogenase lipoamide kinase isozyme 4 (*Pdk4*) was suppressed in *ob/ob* mice compared with normal mice (Figure 3A,B). UDCA significantly reduced protein levels and mRNA expression of PEPCK and G6Pase and had little effect on the mRNA expression of *Pdk4* in *ob/ob* mice (Figure 3A,B), suggesting UDCA reduces gluconeogenesis in *ob/ob* mice. We next detected that *ob/ob* mice had higher mRNA expression of hepatic pyruvate dehydrogenase alpha 1 (*Pdha1*), glycogen synthase *(Gys) 1* and *Gys2*, and lower mRNA expression of phosphatidylinositol 3-kinase regulatory subunit 1 (*Pik3r1*) than normal mice; these were significantly improved in *ob/ob* mice treated with UDCA, but had no effect on *Gys2* (Figure 3C). We focused on the molecular mechanisms by which UDCA affect glucose metabolism in the liver. 

Moreover, analysis of Notch signaling pathways by Western blotting and qPCR revealed that Notch signaling-related genes were upregulated in liver obtained from *ob/ob* mice compared with both normal and *ob/ob* mice treated with UDCA (Figure 3D,F). We detected both Notch ligands and jagged 1 (*Jag1*); deltas like canonical Notch ligand 1 (*Dll1*) and *Dll3* were induced in *ob/ob* mouse liver, resulting in increased neurogenic locus notch homolog protein 1 (Notch1) protein levels (Figure 3D,E). UDCA decreased the protein level of the Notch intracellular domain (NICD) and Notch1 in liver (Figure 3D). Notch receptor-ligand interactions trigger the downstream responses, including the Notch transcriptional targets, such as the recombining binding protein suppressor of hairless (*Rbpj*), hairy and enhancer of split (*Hes) 1* and *Hes 5* in *ob/ob* mice (Figure 3F). Consistently, UDCA significantly downregulated the Notch target genes, *Rbpj, Hes1, Hes5, G6pase,* and *Pepck* in the liver of *ob/ob* mice. UDCA blockade of hepatic Notch signaling resulted in the inhibition of hepatic glucose; the metabolism was associated with downregulation of gluconeogenic mRNA expression (Figure 3).

### 3.4. UDCA Improves Hepatic Mitochondrial Dysfunction and Biogenesis in ob/ob Mice

**Figure 4:**
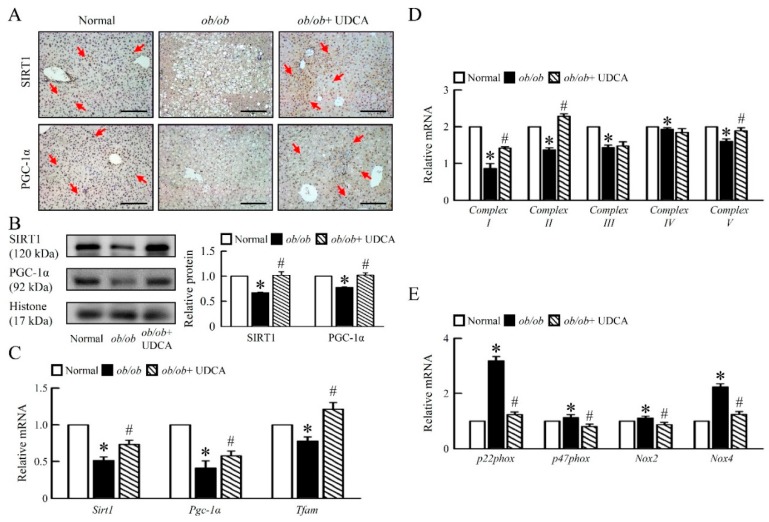
UDCA improves hepatic mitochondria biogenesis and dysfunction in *ob/ob* mice. Normal and *ob/ob* mice were treated with or without 50 mg/kg UDCA for 14 days. (**A**) Immunohistochemical staining analysis of SIRT1 and PGC-1α in the liver. Red arrow highlights the positive staining. Scale bars, 200 µm. (**B**) Western blot analysis of SIRT1 and PGC-1α protein levels in liver. qRT-PCR analysis of (**C**) *Sirt1, Pgc-1α, Tfam*, (**D**) *Complex I, II, III, IV, V,* (**E**) *p22phox, p47phox, Nox2*, and *Nox4* mRNA expression in liver. Relative mRNA expression was normalized to *Gapdh* and then normalized to the controls. In all panels, results are expressed as the mean ± S.E.M. of five independent experiments, and statistical significance of differences between means was assessed using an unpaired Student’s *t*-test (* *p* ≤ 0.05; normal vs. *ob/ob*. ^#^ *p* ≤ 0.05; *ob/ob* vs. *ob/ob* + UDCA). SIRT1, nicotinamide adenine dinucleotide (NAD)-dependent protein deacetylase sirtuin-1; PGC-1α, peroxisome proliferator-activated receptor gamma coactivator 1-alpha; *Tfam*, mitochondrial transcription factors A; *Nox*, nicotinamide adenine dinucleotide phosphate (NADPH) oxidase.

Next, we aimed at understanding the mechanistic underpinnings of the obesity-induced hepatic mitochondrial dysfunction observed in liver tissue of *ob/ob* mice under UDCA treatment. Compared to normal mice, *ob/ob* mice had lower hepatic mitochondrial biogenesis regulators, NAD-dependent deacetylase sirtuin-1 (SIRT1), PPARγ Coactivator-1 (PGC-1α) protein levels (Figure 4A,B), and mitochondrial transcription factor A (*Tfam*) mRNA expression (Figure 4C). 

Further, we showed that *ob/ob* mice had lower basal hepatic mitochondrial *Complex I, II, III, IV,* and *V* mRNA expression than normal mice (Figure 4D). Moreover, treatment with UDCA increased mitochondrial biogenesis regulators; factors; and the mitochondrial *Complex I, II* and *V*, but no effect on mitochondrial *Complex III* and *IV* mRNA expression in *ob/ob* mice (Figure 4D). To identify mechanisms of hepatic mitochondrial dysfunction, we assessed the ROS generation key regulator in obese states. Accordingly, *ob/ob* mice had higher *p22phox, p47phox*, NADPH oxidase *(Nox) 2,* and *Nox4* mRNA expression than normal mice, and UDCA treatment completely inhibited the effects (Figure 4E), suggesting that UDCA ameliorates hepatic mitochondrial dysfunction and inflammation in *ob/ob* mice.

### 3.5. Effects of UDCA on Regulates Bile Acid Metabolism and Alters Transporters Expression in ob/ob Mice

**Figure 5:**
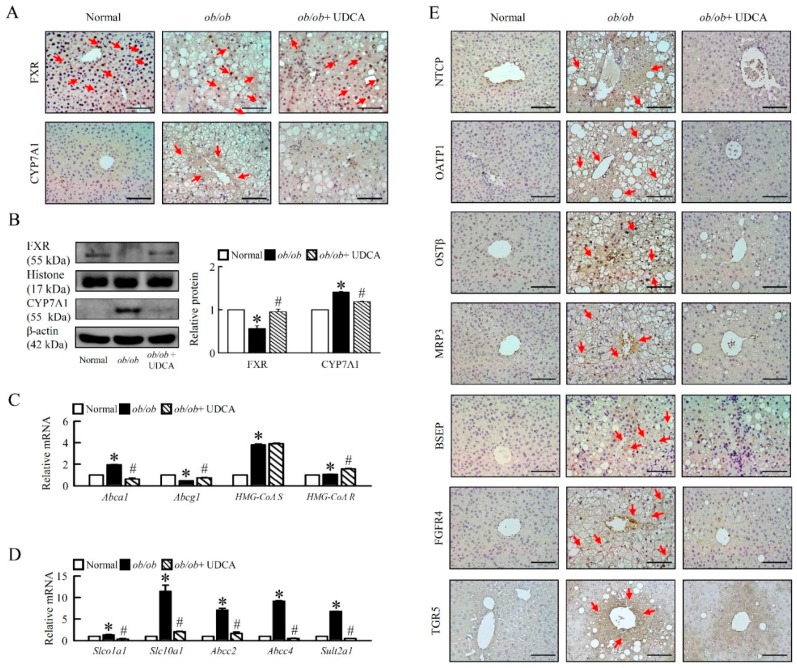
Effects of UDCA regulates bile acid transporters and metabolism in *ob/ob* mice. Normal and *ob/ob* mice were treated with or without 50 mg/kg UDCA for 14 days. (**A**) Immunohistochemical staining analysis of FXR, CYP7A1 in the liver. Red arrow highlights the positive staining. Scale bars, 200 µm. (**B**) Western blot analysis of FXR and CYP7A1 protein levels in liver. qRT-PCR analysis of (**C**) *Abca1, Abcg1, HMG-CoA synthase, HMG-CoA Reductase*, (**D**) *Slco1a1, Slc10a1, Abcc2, Abcc4,* and *Sult2a1* mRNA expression in liver. Relative mRNA expression was normalized to *Gapdh* and then normalized to the controls. (**E**) Immunohistochemical staining analysis of NTCP, OATP1, OSTβ, MRP3, BSEP, FGFR4, and TGR5 in the liver. Red arrow highlights the positive staining. Scale bars, 200 µm. In all panels, results are expressed as the mean ± S.E.M. of five independent experiments, and statistical significance of differences between means was assessed using an unpaired Student’s *t*-test (* *p* ≤ 0.05; normal vs. *ob/ob*. ^#^ *p* ≤ 0.05; *ob/ob* vs. *ob/ob* + UDCA). CYP7A1, cholesterol 7 alpha-hydroxylase; Oatp1 *(Slco1a1)*, organic anion-transporting polypeptide; Ntcp (*Slc10a1*), Na^+^/taurocholate cotransporter; Mrp *(Abcc)*, multidrug-resistance protein; *Sult2a1*, sulfotransferase family 2A member 1; *Abc*, ATP-binding cassette sub-family; *HMG-CoA*, 3-hydroxy-3-methylglutaryl (Hydroxymethylglutaryl)-coenzyme A; BSEP, bile salt export pump, FGFR4, fibroblast growth factor receptor 4; TGR5, G-protein-coupled bile acid receptor 1 (GPBAR1); OSTβ, organic solute transporters.

Based on the observation of increased bile acids levels in plasma and liver of *ob/ob* mice (Table 3), we investigated whether bile acid regulator and transporters are altered in liver from *ob/ob* mice treatment of UDCA. Indeed, a reduced level of FXR and an increased level of cholesterol 7 alpha-hydroxylase (CYP7A1) protein in liver and UDCA reverses these changes in *ob/ob* mice (Figure 5A, B), but there is no effect of plasma and the hepatic concentrations of bile acid (Table 3). To explore the mechanisms whereby disrupting hepatic CYP7A1 normalizes bile acids homeostasis, we assessed plasma and hepatic cholesterol and cholesterol transporters in *ob/ob* mice (Table 3, Figure 5C). Levels of plasma cholesterol and mRNA expression of hepatic ATP-binding cassette transporter A1 *(Abca1)*, 5-hydroxy-3-methylglutaryl-coenzyme A synthase (*HMG-CoA S*), and *HMG-CoA* reductase (R) were higher, and mRNA expression of hepatic ATP-binding cassette sub-family G member 1 *(Abcg1)* was lower in *ob/ob* mice compared with normal mice. UDCA improved plasma cholesterol, hepatic *Abca1* and *Abcg1*, and increased *HMG-CoA R*, but had no effect on *HMG-CoA S* mRNA expression in *ob/ob* mice (Figure 5C). Indeed, detoxification-related enzyme, sulfotransferase family 2A member 1 *(Sult2a1)*, and hepatic bile acid transporters, organic anion-transporting polypeptide (OATP1, *Slco1a1*), Na^+^-taurocholate cotransporting polypeptide (NTCP, *SLC10A1*), organic solute transporters (OSTβ), multidrug resistance-associated protein 3 (MRP3, *Abcc3*), and bile salt export pump (BSEP) protein levels were significantly increased in *ob/ob* mice, and UDCA reduced hepatic bile acid transporters in liver (Figure 5D,E). 

Accordingly, G-protein-coupled bile acid receptor 1 (GPBAR1, TGR5) and fibroblast growth factor receptor 4 (FGFR4) were higher in obese mice, and UDCA significantly reduced FGFR4 but did not significantly reduce TGR5 in obese mice (Figure 5E), suggesting UDCA may have remodified bile acid metabolism by modifying the function of bile acid transporters.

### 3.6. UDCA Attenuates Hepatic Inflammation and Alternative Macrophage Activation in ob/ob Mice

**Figure 6:**
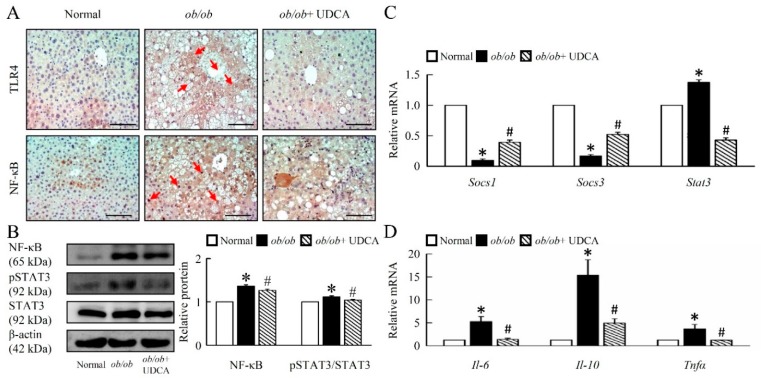
UDCA decreases TLR4 and NF-κB signaling pathways-mediated inflammation in *ob/ob* mice. Normal and *ob/ob* mice were treated with or without 50 mg/kg UDCA for 14 days. (**A**) Immunohistochemical staining of TLR4 and NF-κB in the liver. Red arrow highlights the positive staining. Scale bars, 200µm. (**B**) Western blot analysis of NF-κB, p-STAT3, and STAT3 protein levels in liver. qRT-PCR analysis of (**C**) *Socs1, Socs3*, *Stat3*, (**D**) *Il-6, Il-10,* and *Tnfα* mRNA expression in liver. In all panels, results are expressed as the mean ± S.E.M. of five independent experiments, and statistical significance of differences between means was assessed using an unpaired Student’s *t*-test (* *p* ≤ 0.05; normal vs. *ob/ob*. ^#^ *p* ≤ 0.05; *ob/ob* vs. *ob/ob* + UDCA). TLR4, Toll-like receptors 4; NF-κB, nuclear factor kappa-light-chain-enhancer of activated B cells; p-STAT3, phosphorylation signal transducer and activator of transcription 3; *Socs*, suppressor of cytokine signaling; *Il*, interleukin; *Tnf*, tumor necrosis factor.

To determine the extent of inflammation in the livers of obese mice, we investigated the possible involvement of Toll-like receptor (TLR) 4, NF-κB (Figure 6A,B), and phospho-signal transducer and activator of transcription (p-STAT) 3 in the obese models switching effect of UDCA (Figure 6B). These transcription factors are known to play a crucial role in insensitivity or resistance to mitochondrial-mediated inflammation. UDCA significantly attenuated TLR4, NF-κB, p-STAT3 protein level, and increased *Socs1* and *Socs3* mRNA expression in *ob/ob* mice (Figure 6A–C). Moreover, UDCA significantly reduced hepatic proinflammatory cytokine interleukin (*Il*)-10, M1 markers *Il-6*, and tumor necrosis factor *(Tnf) α* mRNA expression in *ob/ob* mice (Figure 6D). To further estimate the impact of *ob/ob* on liver tissue inflammation, we assessed the protein levels of neutrophil and macrophage M1 and M2 markers. 

**Figure 7:**
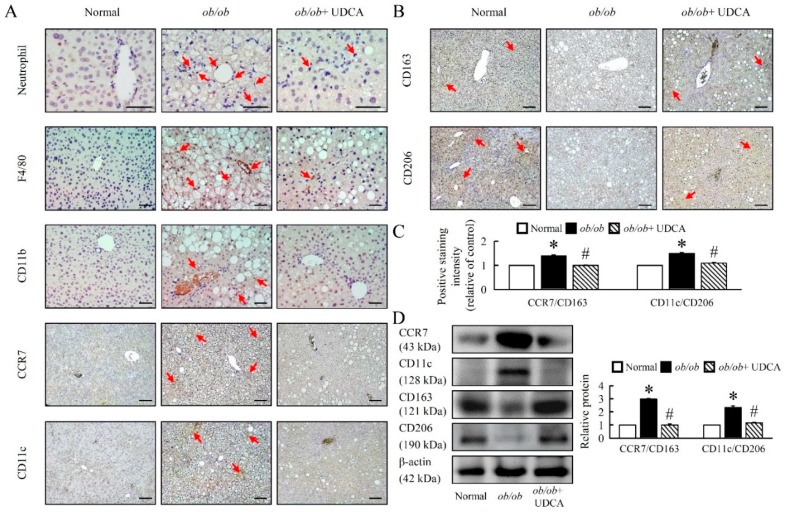
UDCA regulates macrophage M1 and M2 polarization in *ob/ob* mice. Normal and *ob/ob* mice were treated with or without 50 mg/kg UDCA for 14 days. Immunohistochemical staining of (**A**) Neutrophil, F4/80, CD11b, CCR7, CD11c, (**B**) CD163, and CD206 in the liver. Red arrow highlights the positive staining. Scale bars, 100µm. (**C**) The intensity of positive staining (brown color) was measured. (**D**) Western blot analysis of CCR7, CD11c, CD163, and CD206 protein levels in liver. In all panels, results are expressed as the mean ± S.E.M. of five independent experiments, and statistical significance of differences between means was assessed using an unpaired Student’s *t*-test (* *p* ≤ 0.05; normal vs. *ob/ob*. ^#^ *p* ≤ 0.05; *ob/ob* vs. *ob/ob* + UDCA). CD, cluster of differentiation; CCR7, C-C chemokine receptor type 7.

Here, the *ob/ob* mice showed a significant up-regulation of neutrophil, F4/80, CD11b, and classical M1 polarization markers (CCR7 and CD11c), and down-regulation of M2 polarization markers (CD163 and CD206) in *ob/ob* mice (Figure 7A–D). UDCA significantly attenuated M1 markers, increased M2 macrophages markers, and attenuated the ratio of M1 to M2 macrophages (CCR7/CD163 and CD11c/CD206) in *ob/ob* mice (Figure 7C,D). The beneficial effects of UDCA on M2 activation are associated with improved inflammatory conditions in obesity-induced diseases.

### 3.7. UDCA Partially Ameliorates Inflammation-Induced Hepatic Angiogenesis in ob/ob Mice

**Figure 8:**
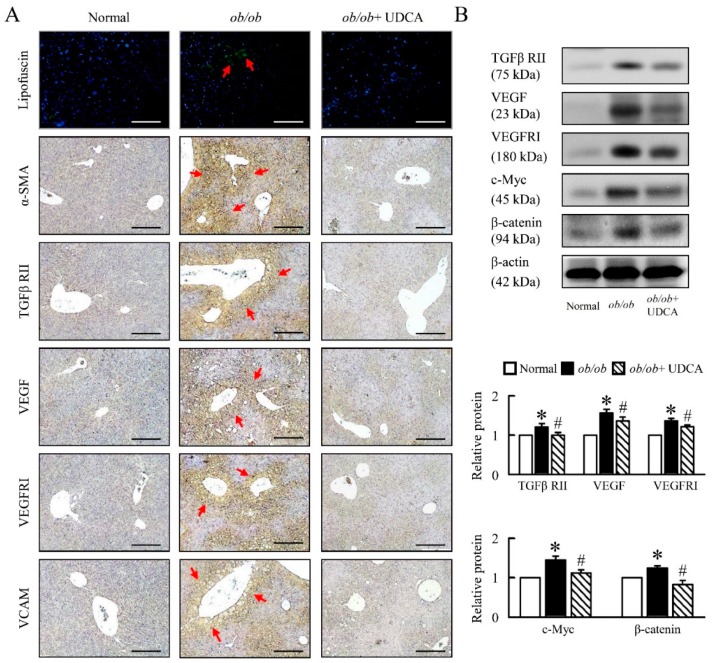
UDCA ameliorates liver lipofuscin and angiogenesis in *ob/ob* mice. Normal and *ob/ob* mice were treated with or without 50 mg/kg UDCA for 14 days. (**A**) Representative pictures of lipofuscin of liver section. Green pseudo-color represents visualization of lipofuscin’s autofluorescence at 450–490 nm. Immunohistochemical staining of α-SMA, TGFβ RII, VEGF, VEGFRI, and VCAM. Red arrow highlights the positive staining. Scale bars, 200µm. (**B**) TGFβ RII, VEGF, VEGFRI, c-Myc, and β-catenin were detected by Western blot analysis in the liver. In all panels, results are expressed as the mean ± S.E.M. of five independent experiments, and statistical significance of differences between means was assessed using an unpaired Student’s *t*-test (* *p* ≤ 0.05; normal vs. *ob/ob*. ^#^ *p* ≤ 0.05; *ob/ob* vs. *ob/ob* + UDCA). α-SMA, alpha-smooth muscle actin; TGFβ RII, transforming growth factor beta receptor II; VEGF, vascular endothelial growth factor; VEGFRI, VEGF receptor I; VCAM, vascular cell adhesion molecule.

Next, we aimed at understanding the mechanistic underpinning of the inflammation and oxidative stress-driven angiogenesis observed in *ob/ob* mice. *ob/ob* mice had higher levels of lipofuscin, alpha-smooth muscle actin (α-SMA), transforming growth factor beta receptor II (TGFβ RII), vascular endothelial growth factor (VEGF), VEGF receptor I (VEGF RI), vascular cell adhesion molecule (VCAM), c-Myc and β-catenin. UDCA significantly reduced liver angiogenesis expression in *ob/ob* mice (Figure 8A,B). These data suggest that UDCA may be important in the regulation of inflammation-induced hepatic angiogenesis in *ob/ob* mice.

### 3.8. UDCA Alters EWAT M1 and M2 Macrophage Polarization and Ameliorates Initial Angiogenesis in ob/ob Mice

**Figure 9:**
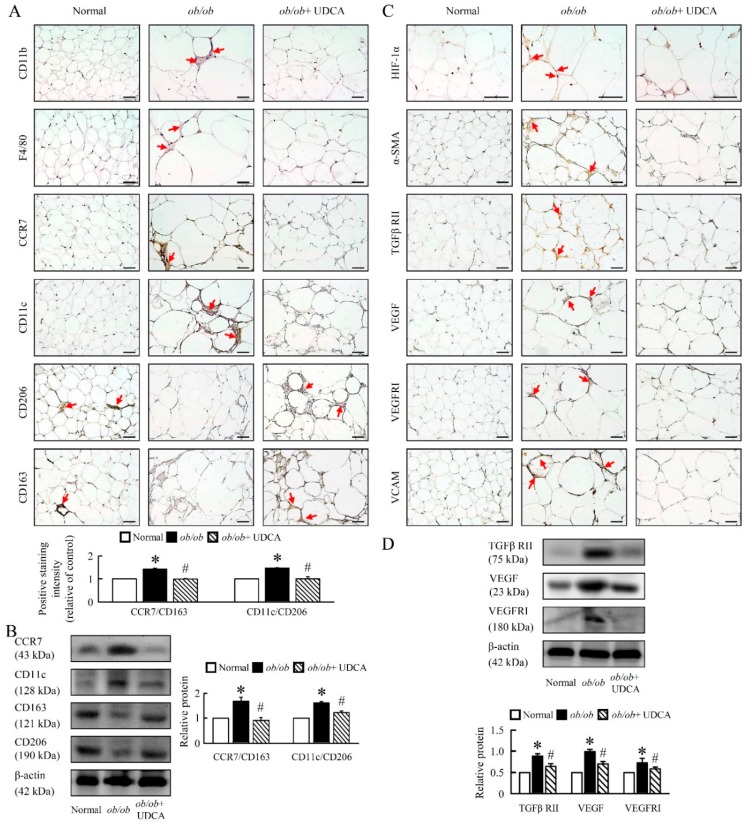
UDCA ameliorates EWAT macrophage infiltration and angiogenesis in *ob/ob* mice. Normal and *ob/ob* mice were treated with or without 50 mg/kg UDCA for 14 days. Immunohistochemical staining of (**A**) CD11b, F4/80, CCR7, CD11c, CD206, and CD163. Scale bars, 50 µm. The intensity of positive staining (brown color) was measured. Red arrow highlights the positive staining. Scale bars, 50 µm. (**B**) CCR7, CD11c, CD163, and CD206 were detected by Western blot analysis in EWAT. Immunohistochemical staining of (**C**) HIF-1α, α-SMA, TGFβ RII, VEGF, VEGFRI, and VCAM. Red arrow highlights the positive staining. Scale bars, 50 µm. (**D**) TGFβ RII, VEGF and VEGFRI were detected by Western blot analysis in EWAT. In all panels, results are expressed as the mean ± S.E.M. of five independent experiments, and statistical significance of differences between means was assessed using an unpaired Student’s *t*-test (* *p* ≤ 0.05; normal vs. *ob/ob*. ^#^ *p* ≤ 0.05; *ob/ob* vs. *ob/ob* + UDCA). EWAT, epididymal white adipose tissue; CD, cluster of differentiation; CCR7, C-C chemokine receptor type 7; HIF, hypoxia-inducible factors; α-SMA, alpha-smooth muscle actin; TGFβ RII, transforming growth factor beta receptor II; VEGF, vascular endothelial growth factor; VEGFRI, VEGF receptor I; VCAM, vascular cell adhesion molecule.

In order to analyze whether UDCA is able to affect adipose tissue macrophages activation, protein expression analyses were performed in EWAT of *ob/ob* mice, as defined by macrophage markers. We found that CD11b, F4/80, CCR7, and CD11c protein levels were significantly increased in *ob/ob* mice as compared with normal mice (Figure 9A,B). We also observed that alternatively activated M2 macrophage markers (CD206 and CD163) were downregulated in *ob/ob* mice (Figure 9A,B). UDCA significantly reduced M1 markers, increased M2 markers, and improved the ratio of M1 to M2 (CCR7/CD163 and CD11c/CD206) macrophages in *ob/ob* mice (Figure 9A,B).

Moreover, we evaluated the levels of hypoxia in adipose tissues using hypoxia-inducible factor 1 α (HIF-1α) immunohistology stain, a chemical probe for hypoxia. The HIF-1α content was increased in the EWAT of *ob/ob* mice (Figure 9C). We next analyzed the expression of genes and the secretion of proteins involved in angiogenesis. α-SMA, TGFβ RII, VEGF, VEGFRI, and VCAM protein levels were higher in the EWAT of *ob/ob* mice, and UDCA inhibited HIF-1α and angiogenesis marker protein levels (Figure 9C,D). Therefore, UDCA ameliorated initial angiogenesis possibly by inhibiting HIF-1α and activating M2 macrophages.

### 3.9. UDCA Attenuates Lipogenesis and Induces Browning in EWAT of ob/ob Mice

**Figure 10:**
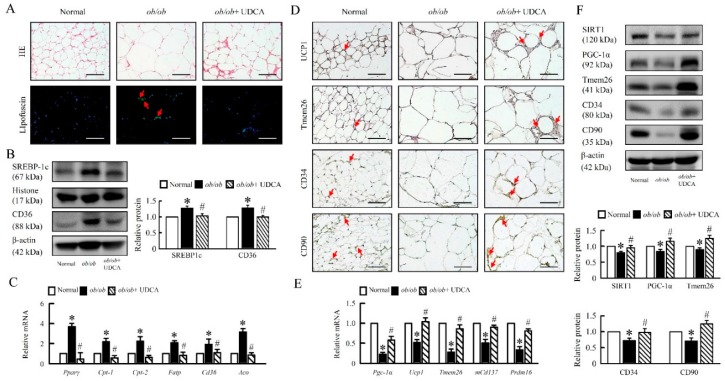
UDCA promotes EWAT browning and reduces obesity. Normal and *ob/ob* mice were treated with or without 50 mg/kg UDCA for 14 days. (**A**) Representative pictures of HE staining and lipofuscin of EWAT section. Green pseudo-color represents the visualization of lipofuscin’s autofluorescence at 450–490 nm. Red arrow highlights the positive staining. Scale bars, 100µm. (**B**) SREBP-1c and CD36 were observed by Western blot analysis in the liver. (**C**) mRNA expression of *Pparγ, Cpt-1, Cpt-2, Fatp, Cd36*, and *Aco* in EWAT were detected by RT-qPCR. Immunohistochemical staining of (**D**) UCP1, Tmem26, CD34, and CD90. Red arrow highlights the positive staining. Scale bars, 100µm. (**E**) The mRNA expression of *Pgc-1α, Ucp1, Tmem26, mCd137*, and *Prdm16* in EWAT were detected by RT-qPCR. (**F**) SIRT1, PGC-1α, Tmem26, CD34, and CD90 were detected by Western blot analysis in EWAT. In all panels, results are expressed as the mean ± S.E.M. of five independent experiments, and statistical significance of differences between means was assessed using an unpaired Student’s *t*-test (* *p* ≤ 0.05; normal vs. *ob/ob*. ^#^ *p* ≤ 0.05; *ob/ob* vs. *ob/ob* + UDCA). EWAT, epididymal white adipose tissue; SREBP-1c, sterol regulatory element-binding proteins; *Pparγ*, peroxisome proliferator-activated receptor γ; *Cpt-1*, carnitine palmitoyltransferase-1; *Fatp*, fatty acid transporter protein; *Aco*, Acyl-CoA oxidase; Tmem26, transmembrane protein 26; *Prdm16*, PR domain zinc finger protein 16.

Lack of leptin led to profound modifications in adipocyte morphology and physiology. Interestingly, these changes were fat depot-specific. EWAT form *ob/ob* mice displayed increased lipofuscin, SREBP-1c, and CD36 protein levels, and *Pparγ, Cpt-1, Cpt-2, Fatp, Cd36, Aco* mRNA expression, suggesting an enhanced lipogenesis, lipid accumulation, and fatty acid oxidation of EWAT (Figure 10A–C). UDCA significantly reduced lipid accumulation and reversed β-oxidation in *ob/ob* mice (Figure 10A–C).

Furthermore, histological analysis of EWAT revealed the presence of adipocytes with reduced cell size and increased multinodular aspect in *ob/ob* mice treatment with UDCA. Consistent with these histological features, protein levels of the thermogenesis markers UCP1, transmembrane protein 26 (Tmem26), SIRT1, and PGC-1α, and mRNA expression of *Pgc-1α, Ucp1, Tmem26, mCd137*, and PR domain containing 16 (*Prdm16*) were significantly upregulated in EWAT of obese mice treat with UDCA as compared to obese mice group (Figure 10D–F). Moreover, UDCA enhanced adipose-derived stem/stromal/progenitor cells (ASCs), CD34, and CD90 in *ob/ob* mice (Figure 10D,F). These results indicate that UDCA induced a distinct coordinated metabolic response that enhances M2 macrophage polarization, ASCs act, and SIRT1-PGC-1α signaling to promote EWAT browning in *ob/ob* mice.

## 4. Discussion

The main important findings on the properties of UDCA arose from the present study: Firstly, the biochemical mechanisms of UDCA in modulating the cross-talk between liver and white adipose tissue regarding bile acid metabolism and fatty acid partitioning in obese mice. Secondly, UDCA activated the SIRT1-PGC1-α signaling pathway and reduced Sterol regulatory element-binding protein-1 (SREBP-1), a key transcription factor that regulates genes in de novo lipogenesis and glycolysis pathways. These findings suggest that UDCA increases the gene expression associated with lipogenesis, and gluconeogenesis induces the down-regulation of intracellular ROS levels and is more effective at reducing body weight gain in obesity mice. Thirdly, UDCA blockade of the Notch1 inflammatory response improves macrophage infiltration and is directly involved in the crosstalk of inflammatory and metabolic pathways. Finally, UDCA is sufficient to induce fat differentiation, including mitochondrial biogenesis and respiratory uncoupling (*PPARγ,* *UCP1, and* *PGC-1α*). 

UDCA is currently used as a ‘panacea’ for pharmacological treatment of a wide range of hepatobiliary disorders. Dysregulated bile acid (BA) metabolism is an important indicator in the pathology of NAFLD, which could progress into more severe forms of liver injury, including lipid, glucose metabolism, immune response, and mitochondrial dysfunction [27,28]. 

Previous studies showed a considerable increase in cholesterol and BA synthesis in obesity [29]. We found that UDCA changes BA and cholesterol synthesis via induction of several BA and cholesterol synthetic markers and enzymes such as FXR and CYP7A1 and hepatic SREBP1 and TGR5 in obese mice. Since diminished cholesterol synthesis in turn decreased BA formation, no net effect on hepatic cholesterol levels was observed. UDCA stimulates CYP7A1 via suppressed hepatic FXR and SREBP-1 signaling pathways. Therefore, UDCA, which constitutes the amount of BAs, then has only a low affinity for and hence no agonistic activity on FXR. Moreover, our data suggest the adaptive changes in bile acid transporters expression in liver provide alternative excretory pathways for biliary constituents during UDCA treatment and may thus attenuate liver injury in *ob/ob* mice. Changes in bile acid transporters constitute a central element in the pathogenesis and clinical manifestation of cholestasis, and several clinically effective drugs modulate transporter expression via regulatory nuclear receptors (NRs), translating into a reduction of the hepatocellular bile acid burden, thus ameliorating tissue injury [30]. Interestingly, we note that UDCA reduced cholesterol synthesis and bile acid transporter levels, but did not reduce the plasma bile acid level, suggesting that UDCA may induce slightly different programs, highlighting the complexity of bile acid signaling.

SIRT1 is an NAD-dependent deacetylase and has been reported to have many beneficial effects for controlled glucose homeostasis, lipid metabolism, and insulin resistance in the liver tissue of obese mice [31]. PGC1-α is regulated by SIRT-1 according to cellular energy requirements and works as a regulator of mitochondrial biogenesis [32]. SIRT1-deficient mice lack AMPK activity, but SREBP-1c expression is promoted in mice with induced obesity and hepatic steatosis compared to wild-type HFD-induced obese mice [33]. Our study has found that UDCA can activate SIRT-1 and reduce hepatic steatosis by increasing fatty acid β-oxidation and reducing SREBP-1c expression. Therefore, sirt1-PGC1-SREBP pathway activity potentially inhibits lipid accumulation in hepatocytes and improves hepatic steatosis. Notably, our study showed that UDCA elevates hepatic and adipose tissue levels of SIRT1, PGC1-α, and simultaneously decreases SREBP-1 in fat mice. Interestingly, accelerating lipid decomposition is an important strategy for improving NAFLD. In the liver, excessive lipid biosynthesis is mainly a phenomenon of excessive energy accumulation. Notably, excess TGs in the liver are broken down, and released free fatty acids must be decomposed to produce energy via fatty acid β-oxidation and reduce the damage to liver and adipose tissue. To our knowledge, this is the first study of UDCA which improves mitochondria function in liver and adipose tissue. UDCA appears to induce adaptive thermogenesis via the SIRT-1-PGC-1α signaling pathway in WAT when mice are challenged with fat accumulation. This adaptive thermogenesis does act through the same signaling pathway in hepatic steatosis. In addition, adaptive thermogenesis in adipose tissue occurs through an increase in mitochondrial mass to accommodate increased metabolic demands for respiratory uncoupling. Importantly, UDCA induces a marked elevation of SIRT-1-PGC-1α in adipose tissues and may indicate that UDCA improves WAT and liver mitochondrial capacity in obese mice.

We have provided compelling genetic, physiological, metabolic, histological, cellular, and molecular evidence to demonstrate that blockage of Notch signaling promotes browning of WAT. White to brown fat transition involves a series of cellular processes, including increased UCP1 expression and mitochondria production, lipolysis, and β-oxidation. We found that obesity increased Notch activity in liver and WAT. These results indicate that the activation of Notch signaling is linked to the initiation and development of obesity. Treating obese mice with UDCA reduced body weight and ameliorated glucose metabolism—such a drastic effect may be elicited not only by browning of white adipose tissue, but also through targeting other important metabolic organs, including liver, which directly regulates lipid metabolism and nutrient absorption respectively. Previous studies have demonstrated that Notch gain-of-function causes fatty liver, and is correlated with an increasing rate of SREBP-1c-mediated lipogenesis and increases hepatic glucose production by co-activating Foxo1 at the G6Pase and PEPCK, and it is regulated in response to metabolic stimuli in the liver [34]. Moreover, liver-specific REPJ deletion protects from diet-induced steatosis, reduces de novo lipogenesis, improves glucose tolerance, and reduces hepatocyte glucose production in mice [34,35]. Here, UDCA led to reducing lipogenesis, gluconeogenesis, and Notch 1 signaling proteins in *ob/ob* mice, suggesting that UDCA plays an important role in lipid, glucose metabolism, and energy storage capacity in those with obesity. 

Moreover, Notch could reprogram for proinflammatory macrophage activation [36]; mitochondrial abnormalities and abnormal morphologic changes associated with NAFLD include decreased activity of respiratory chain complexes, impaired mitochondrial β-oxidation ultrastructural lesions, as well as depletion of mtDNA that have been observed in patients and animal models with NASH [37,38]. The mitochondrial dehydrogenase activities related to complex I (NADH-dehydrogenase) and complex II (succinate dehydrogenase) were decreased in the diabetic rats that were normalized in the UDCA-treated animals [39]. In this report, we have shown that UDCA reverses obesity-reduced SIRT1, PGC1α, and *Tfam* expression, that also increased mitochondria complex I, II, V mRNA expression in *ob/ob* mice, suggesting UDCA is a potential agent to promote mitochondrial biogenesis and improved mitochondrial function. Mitochondria are a target for ROS produced by NADPH oxidase (NOX) and their associated regulatory subunits such as p22phox and p47phox, which are a significant source of ROS, which under certain conditions may stimulate NADPH oxidases [40,41]. Our study reveales that UDCA significantly improved obesity-induced mitochondrial dysfunction by suppressing NOX family enzymes mRNA expression and Notch-mediated lipogenesis, gluconeogenesis in *ob/ob* mice.

Fatty acids released from hypertrophied adipocytes themselves serve as ligands for TLR 4, which is expressed on adipocytes and macrophages and can also provoke inflammation in liver and adipose tissue [42]. Importantly, downregulation of TLR4, NF-κB, and STAT3 signals in *ob/ob* mice after treat with UDCA. Our study showed that the expression of M2 macrophage [43] markers (CD206 and CD163) were down-regulated in the liver and EWAT biopsy samples of the obese subjects. Recent studies also showed that CCR7(-/-) mice were protected from fatty liver and dyslipidemia and exhibited increased thermogenesis on high-fat feeding [44]. By analyzing the expression of genes and the angiocrine factor secretion profiles of different subsets of macrophages, Spiller et al. showed that M1 macrophages express genes involved in the initiation of angiogenesis, including VEGF-A and FGF2 [45]. Thereby, considering that obesity induces a phenotypic switch from an anti-inflammatory M2 polarized state to a proinflammatory M1 state, increased M1 macrophages along with pro-inflammatory cytokines and chemokines contribute to chronic inflammation [46]. Thus, our findings may support the notion that the M2 macrophage polarization plays a prominent role in *ob/ob* mice treat with UDCA.

In accordance with previous observations, adipose tissue has a key role in peripheral energy homeostasis, as obesity is also associated with dysfunctional adipose tissue and fatty liver [47,48]. In our study, we observed that UDCA treatment induced important changes at the level of SIRT-1-PGC-1 and SREBP-1 signaling involved in lipid metabolism and energy in adipose tissue. These data accord with results that UDCA affects energy balance by promoting a profound remodeling in lipid-specific adipocyte functions. These changes were associated with enhanced mitochondrial function and increased M2 macrophage activation in liver and EWAT. Thus, in agreement with previously published evidence [49], UDCA has the effect of reducing the lipid storage capacity of liver and WAT. The reduced energy storage capacity was linked to decreased body weight in *ob/ob* mice. Moreover, protein levels on analysis confirmed upregulation of thermogenic genes and mitochondrial biogenesis in EWAT, suggesting the browning of this fat depot in *ob/ob* mice treatment with UDCA. Furthermore, adipose-derived stem/stromal/progenitor cells (ASCs), CD34, and CD90 were markedly enhanced in the *ob/ob* mice treatment with UDCA. Altogether, UDCA was able to increase the number of multilocular UCP1 and Tmem26-positive adipocytes in EWAT, therefore inducing EWAT thermogenesis in *ob/ob* mice. UDCA promotes profound energy metabolism, not only of liver, but also of EWAT, including alternatively activated macrophages that work concomitantly with mitochondrial function effects to play a pivotal role in increased stem cells activation and browning EWAT as well as a thermogenic program. In contrast, our current experimental approaches cannot directly estimate the contribution of alternatively activated macrophages in comparison with UDCA to the enhancement of mitochondrial function observed in *ob/ob* mice, or determine whether the M2 macrophages are local sources of sufficient mitochondrial concentrations with physiological relevance. In summary, we conclude that UDCA in liver and EWAT leads to an increased mitochondrial function and M2 macrophage abundance, both of which play critical roles in the emergence of the *ob/ob* mice. This compelling evidence illustrates the crosstalk between adipocytes and immune cells in concert with the liver and EWAT, in which UDCA occupies a key regulatory function.

## Figures and Tables

**Table 1:** Antibodies list.

Antibody	Number	Company	Dilution
α-SMA	A5228	Sigma, USA	IHC 1:80
β-actin	MAB1501	Millipore, Germany	WB 1:1000
β-catenin	ab6302	Abcam, UK	WB 1:500
BSEP	ab217532	Abcam, UK	IHC 1:80
c-Myc	ab32	Abcam, UK	WB 1:500
CCR7	ab1657	Abcam, UK	WB 1:500; IHC 1:80
CD11b	ab133357	Abcam, UK	IHC 1:80
CD11c	ab11029	Abcam, UK	IHC 1:80
CD11c	ab52632	Abcam, UK	WB 1:500
CD163	ab182422	Abcam, UK	WB 1:500; IHC 1:80
CD206	ab64693	Abcam, UK	WB 1:500; IHC 1:80
CD34	ab8158	Abcam, UK	WB 1:500; IHC 1:80
CD36	SC-70644	Santz cruz, USA	WB 1:500; IHC 1:80
CD36, Alexa Fluor 647	102609	BioLegend, USA	IF 1:100
CD90	ab3105	Abcam, UK	WB 1:500; IHC 1:80
CYP7A1	SC-518007	Santz cruz, USA	WB 1:500; IHC 1:80
F4/80	123102	BioLegend, CA	IHC 1:80
FGFR4	ab44971	Abcam, UK	IHC 1:80
FXR	SC-13063	Santz cruz, USA	WB 1:500; IHC 1:80; IF 1:80
G6Pase	SC-25840	Santz cruz, USA	WB 1:500
HIF-1α	ab1	Abcam, UK	IHC 1:60
Histone	SC-56616	Santz cruz, USA	WB 1:1000
MRP3	ab3375	Abcam, UK	IHC 1:80
Neutrophil	ab2557	Abcam, UK	IHC 1:80
NF-κB	ab16502	Abcam, UK	WB 1:500; IHC 1:80; IF 1:80
Notch1	ab52627	Abcam, UK	WB 1:500
NICD	ab8925	Abcam, UK	WB 1:500
NTCP	ab131084	Abcam, UK	IHC 1:80
OATP1	ab203036	Abcam, USA	IHC 1:80
OSTβ	orb1964	Biorbyt, USA	IHC 1:80
PEPCK	SC-377027	Santz cruz, USA	WB 1:500
PGC-1α	ab54481	Abcam, UK	WB 1:500; IHC 1:80
SIRT1	ab110304	Abcam, UK	WB 1:500; IHC 1:80
SREBP-1c	SC-366	Santz cruz, USA	WB 1:500; IHC 1:100; IF 1:80
p-STAT3	05-485	Millipore, Germany	WB 1:500
STAT3	06-596	Millipore, Germany	WB 1:500
TGFβ RII	SC-17791	Santz cruz, USA	WB 1:500; IHC 1:100
TGR5	ab72608	Abcam, UK	IHC 1:80
TLR4	ab13556	Abcam, UK	IHC 1:80
Tmem26	ab186640	Abcam, UK	WB 1:500; IHC 1:80
UCP1	ab10983	Abcam, UK	WB 1:500; IHC 1:80
VCAM	SC-8304	Santz cruz, USA	IHC 1:80
VEGF	SC-80434	Santz cruz, USA	IHC 1:80
VEGF	ab69479	Abcam, UK	WB 1:500
VEGFRI	1303-1	Epitomics, USA	WB 1:500; IHC 1:80
DAPI	62248	Thermo Fisher Scientific, USA	IF 1:10000
Mouse Secondary Antibody	G-21040	Thermo Fisher Scientific, USA	WB 1:3000; IHC 1:100
Rabbit Secondary Antibody	G-21234	Thermo Fisher Scientific, USA	WB 1:3000; IHC 1:100
Goat Secondary Antibody	31402	Thermo Fisher Scientific, USA	WB 1:3000; IHC 1:100
Rat Secondary Antibody	31470	Thermo Fisher Scientific, USA	WB 1:3000; IHC 1:100
Mouse Secondary Antibody, Alexa Fluor 488	A-11029	Thermo Fisher Scientific, USA	IF 1:100
Rabbit Secondary Antibody, Alexa Fluor 488	A11034	Thermo Fisher Scientific, USA	IF 1:100
Rabbit Secondary Antibody, Alexa Fluor Plus 647	A32795	Thermo Fisher Scientific, USA	IF 1:100

α-SMA, α-smooth muscle actin; BSEP, bile salt export pump; CCR7, C-C chemokine receptor type 7; CD11b, cluster of differentiation 11b; CYP7A1, cholesterol 7 alpha-hydroxylase; FGFR4, fibroblast growth factor receptor 4; FXR, farnesoid X receptor; G6Pase, glucose 6-phosphatase; HIF-1α, hypoxia-inducible factor-1α; MRP3, multidrug resistance-associated protein 3; NF-κB, nuclear factor kappa-light-chain-enhancer of activated B cells; Notch1, neurogenic locus notch homolog protein 1, NICD, Notch intracellular domain; NTCP, Na+-taurocholate cotransporting polypeptide; OATP1, organic anion-transporting polypeptide 1; OSTβ, organic solute transporter β; PEPCK, phosphoenolpyruvate carboxykinase; PGC-1α, peroxisome proliferator-activated receptor gamma coactivator-1α; SIRT1, NAD-dependent deacetylase sirtuin 1; SREBP-1c, sterol regulatory element-binding protein-1c; STAT3, signal transducer and activator of transcription 3; TGFβ RII, transforming growth factor beta receptor II; TGR5, G-protein-coupled bile acid receptor 1, TLR4, Toll-like receptors 4; Tmem26, transmembrane protein 26; UCP1, uncoupling protein 1; VCAM, vascular cell adhesion molecule; VEGF, vascular endothelial growth factor; VEGFRI, VEGF receptor I; DAPI, 4’,6-diamidino-2-phenylindole; IHC, immunohistochemistry; IF: immunofluorescence; WB, Western blot.

**Table 2:** Oligonucleotide sequences for-qPCR.

Gene	Forward	Reverse
*SREBP-1c*	5′ actgtcttggttgttgatgagctggagcat 3′	5′ atcggcgcggaagctgtcggggtagcgtc 3′
*FAS*	5′ tgtcattggcctcctcaaaaagggcgtcca 3′	5′ tcaccactgtgggctctgcagagaagcgag 3′
*SCD-1*	5′ ccggagaccccttagatcga 3′	5′ tagcctgtaaaagatttctgcaaacc 3′
*FATP*	5′ gcttcaacagccgtatcctc 3′	5′ tcttcttgttggtggcactg 3′
*CD36*	5′ gcaaaacgactgcaggtcaac 3′	5′ tggtcccagtctcatttagcca 3′
*CPT-1*	5′ ggacagagactgtgcgttcct 3′	5′ gcgatatccaacagtgcttga 3′
*CPT-2*	5′ caaggccctggctgatgatgtg 3′	5′ agtctctgtccgcccctctcg 3′
*ACO*	5′ atgaatcccgatctgcgcaaggagc 3′	5′ aaaggcatgtaacccgtagcactcc 3′
*PPARα*	5′ cgtacggcaatggctttatc 3′	5′ aacggcttcctcaggttctt 3′
*PPARγ*	5′ cctcaaacttggcaatactc 3′	5′- agcaacaacataagcgtcat 3′
*Ppargc1b*	5′ cagccagtacagccccgatg 3′	5′ ggtgtgtcgccttcatccag 3′
*ATGL*	5′ aacaccagcatccagttcaa 3′	5′ ggttcagtaggccattcctc 3′
*HSL*	5′ agacaccagccaacggatac 3′	5′ catcaccctcgaagaagagca 3′
*DGAT*	5′ tcctgaattggtgtgtggtg 3′	5′ ggcgcttctcaatctgaaat 3′
*LCAD*	5′ tcaacagcagttacttgg 3′	5′ gacaatatctgagtggag 3′
*LPL*	5′ actcatctccgccatgcc 3′	5′ ccagctttctcctagcaagg 3′
*ACADM*	5′ ggggaggatgacggagcagc 3′	5′ cgggtactttaggatctggg 3′
*Notch1*	5′ ccagcatggccagctctgg 3′	5′ catccagatctgtggccctgtt 3′
*Jag1*	5′ gtccacggcacctgcaatg 3′	5′ caaggtttggcctcgcact 3′
*RBPJ*	5′ tggcactgttcaatcgcctt 3′	5′ aatcttgggagtgccatgcca 3′
*Dll1*	5′ actccttcagcctgcctga 3′	5′ tatcggatgcactcatcgc 3′
*Dll3*	5′ ctggtgtcttcgagctaca 3′	5′ acacgtgctagcaggttcc 3′
*Hes1*	5′ aaagacggcctctgagcaca 3′	5′ tcatggcgttgatctgggtca 3′
*Hes5*	5′ aagtaccgtggcggtggagatgc 3′	5′ cgctggaagtggtaaagcagctt 3′
*PEPCK*	5′ agcctgctccagctttga 3′	5′ ccctagcctgttctctgtgc 3′
*G6Pase*	5′ tgctgtgtctggtaggcaac 3′	5′ agaatcctgggtctccttgc 3′
*Pdk4*	5′ gatcctaaccaccgccagcc 3′	5′ gcaaaggacgttctttcacag 3′
*Pdha1*	5′ gccgagtgctggttgcttccc 3′	5′ gtctgcatcatcctgtagtacttgagcc 3′
*Gys1*	5′ cggttgtcggacttgctagattgg 3′	5′ cataggtgaagtggtctggaaaggc 3′
*Gys2*	5′ ccttggggtgtttccatcgtac 3′	5′ cggagaggtttgtagtcacactgg 3′
*Pik3r1*	5′ cccactactgtagccaacaacagc 3′	5′ gagtgtaatcgccgtgcattttag 3′
*IL-6*	5′-gtactccagaagaccagagg-3′	5′-tgctggtgacaaccacggcc-3′
*IL-10*	5′ ctggctcagcactgctat 3′	5′ attcatggccttgtagacac 3′
*TNFα*	5′ ttgacctcagcgctgagttg 3′	5′ cctgtagcccacgtcgtagc 3′
*SOCS1*	5′ gtggttgtggagggtgagat 3′	5′ cccagacacaagctgctaca 3′
*SOCS3*	5′ taggaggcgcagccccaagg 3′	5′ gcggcgggaaacttgctgtg 3′
*STAT3*	5′ cgacccaggtgctgccccgta 3′	5′ atgggggaggtagcacactccga 3′
*SIRT1*	5′ gcaacagcatcttgcctgat 3′	5′ gtgctactggtctcactt 3′
*PGC-1α*	5′ gactcagtgtcaccaccgaaa-3′	5′ tgaacgagagcgcatcctt 3′
*TFAM*	5′ ggaatgtggagcgtgctaaaa 3′	5′-tgctggaaaaacacttcggaata 3′
*UCP1*	5′ cctgcctctctcggaaacaa 3′	5′-tgtaggctgcccaatgaaca 3′
Complex I (20kDa)	5′ ccagctgcgcagagttcatc 3′	5′ gagagagcttggggaccacg 3′
Complex II (Ip)	5′ tctaccgctgccacaccatc 3′	5′ aagccaatgctcgcttctcc 3′
Complex III (Core II)	5′ ccattggaaatgcagaggca 3′	5′ ggctggtgacttcctttggc 3′
Complex IV (Cox2)	5′ tcatgagcagtcccctccct 3′	5′ gccatagaataaccctggtcgg 3′
Complex V (F1α)	5′ atctatgcgggtgtacgggg 3′	5′ agggactggtgctggctgat 3′
*p22phox*	5′ tggcctgattctcatcactgg 3′	5′ gggacaactccacagaaactc 3′
*p47phox*	5′ acatcacaggccccatcatccttc 3′	5′-atggattgtcctttgtgcc 3′
*NOX2*	5′ actccttgggtcagcactgg 3′	5′ gttcctgtccagttgtcttcg 3′
*NOX4*	5′ tgaactacagtgaagatttccttgaac 3′	5′ gacacccgtcagaccaggaa 3′
*Oatp1*	5′ gtcttacgagtgtgctccagat 3′	5′ ggaatactgcctctgaagtggatt 3′
*Ntcp*	5′ caccatggagttcagcaaga 3′	5′ agcactgaggggcatgatac 3′
*Mrp2*	5′ gcttcccatggtgatctctt 3′	5′ atcatcgcttcccaggtact 3′
*Mrp4*	5′ ttagatgggcctctggttct ’	5′ gcccacaattccaaccttt 3′
*Sult2a1*	5′ ggaaggaccacgactcataac 3′	5′ gattcttcacaaggtttgtgttacc 3′
*ABCA1*	5′ tggacatcctgaagccag 3′	5′ ttcttcccacatgccct 3′
*ABCG1*	5′ gctgggaagtccacactc 3′	5′ gatacggcacgagattgg 3′
*HMG Co S*	5′ tatgatggtgtagatgctgggaagtatacc 3′	5′ taagttcttctgtgcttttcatccac 3′
*HMG Co R*	5′ gggacggtgacacttaccatctgtatgatg 3′	5′ atcatcttggagagataaaactgcca 3′
*Tmem26*	5′ accctgtcatcccacagag 3′	5′ tgtttggtggagtcctaaggtc 3′
*CD137*	5′ cgtgcagaactcctgtgataac 3′	5′ gtccacctatgctggagaagg 3′
*Prdm16*	5′ cagcacggtgaagccattc	5′ gcgtgcatccgcttgtg 3′
*GAPDH*	5′ tcaccaccatggagaaggc 3′	5′ gctaagcagttggtggtgca 3′

SREBP-1c, sterol regulatory element-binding protein 1c; FAS, fatty acid synthase, SCD-1, stearoyl-CoA desaturase-1, FATP, fatty acid transport protein; CD36, cluster of differentiation 36; CPT-1, carnitine palmitoyltransferase 1; ACO, acyl-CoA oxidase; PPAR, peroxisome proliferator-activated receptor; ATGL, adipose triglyceride lipase; HSL, hormone-sensitive lipase; DGAT, diacylglycerol acyltransferases; LCAD, Long-chain acyl-CoA dehydrogenase; LPL, lipoprotein lipase; ACADM, medium-chain acyl-CoA dehydrogenase (MCAD); Jag1, Jagged1; RBPJ, recombining binding protein suppressor of hairless; Dll, Delta-like protein; Hes, hairy and enhancer of split; PEPCK, phosphoenolpyruvate carboxykinase; G6Pase, glucose 6-phosphatase; Pdk4, pyruvate dehydrogenase lipoamide kinase isozyme 4; Pdha1, pyruvate dehydrogenase alpha 1; Gys, glycogen synthase; Pik3r1, phosphatidylinositol 3-kinase regulatory subunit alpha; IL-6, interleukin-6; TNFα, tumor necrosis factor α; SOCS1, suppressor of cytokine signaling 1; STAT3, signal transducer and activator of transcription 3; SIRT1, NAD-dependent deacetylase sirtuin-1; PGC-1α, peroxisome proliferator-activated receptor gamma coactivator 1-alpha; TFAM, mitochondrial transcription factor A; UCP1, uncoupling protein 1; NOX1, NADPH oxidase 1; Oatp, organic anion-transporting polypeptide; Ntcp, Na+-taurocholate cotransporting polypeptide; Mrp2, multidrug resistance-associated protein 2; Sult2a1, sulfotransferase family 2A member 1; ABCA1, ATP-binding cassette transporter; ABCG1, ATP-binding cassette sub-family G member 1; HMG Co S, HMG-CoA Synthase; HMG Co R; HMG-CoA Reductase; Tmem26, transmembrane protein 26; GAPDH, glyceraldehyde-3-phosphate dehydrogenase.

**Table 3:** Effects of UDCA on body weight; plasma ALT; and hepatic, TBA, TG, and FFA levels in *ob/ob* mice.

Items	Normal	*ob/ob*	*ob/ob* + UDCA 50
Body weight (g)	27.57 ± 0.74	63.68 ± 3.15 **	55.97 ± 2.09 ^#^
Liver weight/Body weight (%)	4.69 ± 0.29	5.42 ± 0.24 *	4.53 ± 0.17 ^#^
Plasma ALT (IU/dL)	26.87 ± 7.62	111.47 ± 21.40 **	56.93 ± 12.34 ^##^
Plasma TBA (μ mol/L)	27.46 ± 11.36	61.52 ± 3.84 **	57.95 ± 2.77
Plasma TG (mg/dL)	85.26 ± 11.12	157.62 ± 5.30 **	123.15 ± 8.44 ^##^
Plasma FFA (mmol/L)	1.22 ± 0.02	1.29 ± 0.01 **	1.13 ± 0.04 ^##^
Plasma Cholesterol (mmol/L)	58.15 ± 2.93	111.53 ± 12.55 **	76.96 ± 5.77 ^##^
Liver TBA (mmol/g liver)	0.85 ± 0.04	1.02 ± 0.06 **	1.06 ± 0.11
Liver TG (mmol/g liver)	48.18 ± 2.18	111.21 ± 1.39 **	102.02 ± 3.32 ^##^
Live FFA (mmol/g liver)	36.61 ± 0.06	69.71 ± 0.78 **	56.29 ± 1.17 ^##^

In all panels, results are expressed as the mean ± S.E.M. of five independent experiments, and statistical significance of differences between means was assessed using an unpaired Student’s *t*-test (* *p* < 0.05, ** *p* < 0.01; normal vs. *ob/ob*. ^#^ *p* < 0.05, ^##^ *p* < 0.01; *ob/ob* vs. *ob/ob* + UDCA). ALT, alanine aminotransferase; TBA, total bile acid; TG, triglyceride; FFA, free fatty acid.
